# The European Tertiary Education Register, the reference dataset on European Higher Education Institutions

**DOI:** 10.1038/s41597-023-02353-2

**Published:** 2023-07-08

**Authors:** Benedetto Lepori, Agata A. Lambrechts, Daniel Wagner-Schuster, Georg Zahradnik

**Affiliations:** 1 Institute for Communication and Public Policy, Università della Svizzera italiana, Lugano, Switzerland; 2 JOANNEUM RESEARCH Forschungsgesellschaft mbH - POLICIES, Graz, Austria; 3 AIT Austrian Institute of Technology GmbH, Vienna, Austria

**Keywords:** Education, Databases

## Abstract

The European Tertiary Education Register (ETER) is the reference dataset on European Higher Education Institutions (HEIs). ETER provides data on nearly 3,500 HEIs in about 40 European countries, including descriptive information, geographical information, students and graduates (with various breakdowns), revenues and expenditures, personnel, and research activities; as of March 2023, data cover the years from 2011–2020. ETER complies with OECD-UNESCO-EUROSTAT standards for educational statistics; most data are collected from National Statistical Authorities (NSAs) or ministries of participating countries and are subject to extensive checks and harmonization. The development of ETER has been funded by the European Commission and is part of the current efforts to establish a European Higher Education Sector Observatory; it is closely connected to the establishment of a broader data infrastructure in the field of science and innovation studies (RISIS). The ETER dataset is widely used in the scholarly literature on higher education and science policy, as well as for policy reports and analyses.

## Background & Summary

Traditionally, data on Higher Education have been provided at the level of whole countries by statistical authorities and international organizations, such as the OECD and UNESCO, to enable comparison of the performance of national systems in terms of educational attainments or research output^1^. To this aim, a set of standards and classifications to achieve international comparability have been developed, such as the UNESCO-OECD-EUROSTAT manual on educational statistics^2^ (UOE) and the Frascati manual on research and development^3^. These methodologies have constituted the basis for extensive international comparisons based on a common definition of educational levels or research performing sectors^4,5^.

Yet, national systems are composed of individual Higher Education Institutions (HEIs) that are “as different as chalk and cheese”^6^. While US higher education was always characterized by high institutional diversity^7^, most European systems were historically composed of a rather homogeneous set of (PhD awarding) universities under tight state control^8^ and with low autonomy^9^. This changed rapidly from the 1970s onward. On the one hand, higher education systems expanded to include different types of professionally oriented institutions, such as colleges and universities of applied sciences^10^. On the other hand, new governance models inspired by New Public Management^11^ emphasized the strategic autonomy of HEIs^12^ and, accordingly, the notion of competitive and strategic differentiation^13^. Consequently, individual HEIs and their heterogeneity have become a relevant unit/level of analysis^14^.

While the systematic collection of institutional-level data in the US started already in the 1960s and evolved to an integrated data system in the 1980s already^15^, no similar system existed in Europe until recently. Data at the level of individual HEIs were mostly collected for administrative purposes and not always made available to the general public; national fragmentation also made the comparability of national data problematic^16^. Even seemingly simple information, such as the number of HEIs in Europe, was difficult to compute reliably from national data.

Establishment of the European Tertiary Education Register (ETER; http://www.eter-project.com) in 2013, following the feasibility study (EUMIDA, 2009–2010)^17^, aimed at filling this gap. This initiative is supported by the European Commission together with other tools aimed at improving the evidence base for policy-making, analysis and transparency, part of the European agenda for the modernisation of Europe’s higher education systems, such as the U-MULTIRANK comparative tool for universities (https://www.umultirank.org/).

More specifically, today, the ETER infrastructure provides a) a stable list of Higher Education Institutions in Europe, including core descriptive information, such as the legal status, the foundation year and geographical information (register function) and b) a set of quantitative data on core dimensions of HEI’s resources and activities such as financial resources, personnel, educational activities (students and graduates) and research activities (PhD students and participations to European Framework programs). Most data for ETER are provided by NSAs or ministries, complying with the standards of international educational statistics, and further checked for consistency and data quality by the ETER team.

A core feature of ETER is the introduction of stable organizational identifiers for HEIs, which are shared with the OrgReg register of public research organizations developed by the European Research Infrastructure in Science, Research and Innovation RISIS (https://www.risis2.eu/registers-orgreg/)^18^. OrgReg also provides extensive information on linkages between HEIs and other entities, such as associated hospitals, and on demographic events^19^, which are highly relevant to interpret quantitative data^20^. ETER/OrgReg identifiers have been matched with several other relevant identifiers in the field, such as the World Higher Education database identifiers, the European Commission Erasmus Charter codes and internal identifiers used by publication, patent and project datasets linked to the RISIS infrastructure. This correspondence allows further integration of ETER data with data from other European sources, such as Erasmus student mobility data and international datasets on publications and patents.

### ETER usage in scholarly literature and policy reports

In just a few years, the ETER dataset has become widely used in scholarly literature on European higher education; a search in Google Scholar retrieved as of April 2023 more than 300 documents referring to ETER as a data source. While some papers use ETER data directly for their analysis, others either exploit ETER to create a sample for collecting additional data and integrate them with data from ETER to characterize the HEIs studied.

ETER has been used, for example, to analyze the internationalization process of European HEI^21,22^, to study the staff composition of HEIs^23–25^ and to analyze the mobility of students in Europe^26^.

Further, ETER enabled the first systematic insights into the diversity of HEIs^17,27^ and national systems in Europe^28^, paving the way for the first attempts to develop an empirical classification of European HEIs^14^. When combined with similar data from the US and with bibliometric information, ETER aided understanding of the ‘performance gap’ of European HEIs^29^ and enabled demonstrating that the position of HEIs in international rankings is strongly associated with institutional budgets^30^.

Two important areas where ETER data have been used have been the study of the efficiency of HEIs^31^ and that of the contribution of higher education to regional economic development^32^ and to the formation of spin-off companies^33^.

Beyond these broad studies, ETER has also been used to characterize specific groups of HEIs, such as military schools^34^, technical universities^35^ or members of the European Universities initiative^36^, and for specific topics, such as language use^37^ and digitalization of HEIs^38^.

Not less important has been the increasing use of ETER for policy analysis and evaluation undertaken by the European Commission and its contractors on topics such as analyzing academic personnel^39^, measuring the travel distance of citizens to HEIs in Europe^40^, studying higher education funding in Europe^41^, or analyzing determinants of students’ mobility^42^.

These examples of scholarly work and policy reporting utilizing ETER display the breadth of the topics analyzed and, accordingly, emphasize the function of ETER as a basic data infrastructure, which provides core data on HEIs such as their geographical position, size, and the number of students.

## Methods

ETER data collection process involved a Europe-wide collection of secondary data, primarily that processed by national authorities (NSAs or ministries) for the data collection on educational statistics coordinated by the European Statistical Agency EUROSTAT. As discussed below, National Authorities delivered data directly to ETER.

Additionally, the project team has extracted some of the data from institutional Websites and Wikipedia entries of the HEIs included in ETER: this concerns information such as the foundation year, main seat, institutional history.

Since ETER is based on data generated for the data collection on educational statistics, definitions for variables are aligned as much as possible with the UNESCO/OECD/EUROSTAT (UOE) manual on educational statistics^2^. This common set of definitions and standards ensures comparability and consistency across countries and institutions and is codified in a detailed methodological handbook available on Zenodo^43^.

More detailed information on the earlier infrastructure developments and a description of decisions made on design, definition and methodology can be found in^44^.

### The data collection process

**Fig. 1:**
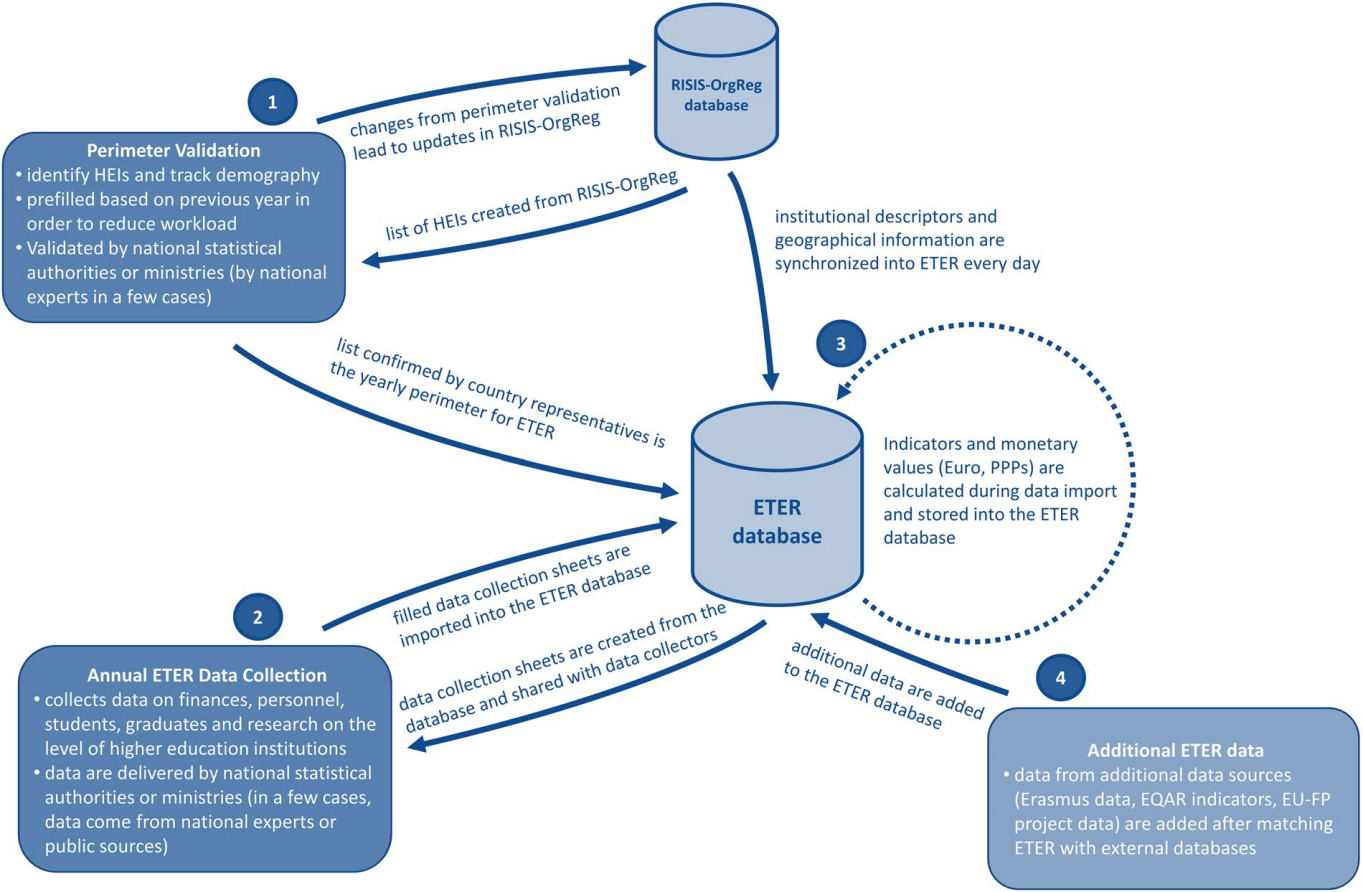
Overview of the ETER data collection process.

Figure 1 gives an overview of the data collection process and its different phases.

#### Perimeter validation

The first step in the data collection was the definition of the perimeter, i.e., the list of institutions to be included in the data collection. Tertiary education is defined in educational statistics by the level of educational programs (International Standard Classification of Educational Degrees ISCED levels 5 to 8). Such programs include professional education as well as doctorate degrees, resulting in several hundreds or thousands of institutions, even for middle-sized countries. However, many tertiary education activities take place in a large number of relatively small institutions, but a major part of higher education, measured in student or graduate numbers, can be found in a smaller number of larger institutions. To make data collection feasible, the ETER project team used delimitation criteria for the inclusion of institutions:

- *major activity*, i.e., education at ISCED levels 6, 7 and/or 8 is a major activity and part of an institution’s mission;
- *graduation* at ISCED levels 6, 7 and/or 8;
- *national recognition as a higher education institution*.

For unclear cases, the following additional criteria were used: (1) size and visibility, i.e., institutions with less than 30 full-time equivalents of academic personnel and less than 200 students could be excluded, except the institution is primarily awarding degrees at ISCED level 8 (leading to a PhD or equivalent award), and (2) continuity, i.e., being a stable organization.

In the perimeter validation process, the ETER project team created perimeter validation files using RISIS-OrgReg as the basis. Basic data include institutional descriptors and geographic information, which are synchronized into ETER on a daily basis. National authorities received a proposed list of institutions from the ETER team, already including demographic events derived from RISIS-OrgReg. National authorities then validated the list by either confirming or adapting the list of institutions and corresponding demographic events. The validated perimeters were used to update RISIS-OrgReg and create the ETER data collection files.

#### The annual ETER data collection

The ETER team created data collection files in MS Excel, which included prefilled information for descriptors, geographic data and other variables which are not likely to change (e.g., lowest, and highest degree delivered). The data deliverer could update prefilled values at any time. The data collection sheets also included extensive control mechanisms. The data collection sheets were then shared with the data deliverer on a cloud-based collaboration platform. There, the data deliverer could access and edit the files. Data were uploaded to the ETER dataset when data collection files were finalized.

#### Indicator and monetary value calculation

Indicators as well as some monetary values were calculated during the import process and stored in the ETER database. Monetary values were delivered in the national currency and converted into Euros and Purchasing Power Parities (PPP) during data import. Monetary conversion and indicators are the only values in the database generated by the ETER project team.

#### Addition of data from external sources

ETER includes data on the institutional level, which can be matched with other institutional datasets. After matching, several of these datasets were added to the ETER dataset and updated manually alongside the main data collection on an annual basis or via an API.

## Data Records

The ETER dataset currently includes nearly 3,500 HEIs in 41 European countries, i.e., EU-27 member states, European Economic Area countries (Iceland, Liechtenstein, and Norway), Switzerland, the UK, and most of the candidate and potential EU candidate countries (North Macedonia, Serbia, Montenegro, Kosovo, Bosnia and Herzegovina, Albania, and Türkiye). Some data is also available for Andorra and the Holy See. Data are collected annually and, as of spring 2023, cover the period 2011–2020 for most countries (with some missing years particularly in the Balkan countries and in France).

As a rule, ETER should include all HEIs in the countries within the perimeter which deliver at least a bachelor’s degree (ISCED level 6), and which exceed a certain size. These restrictions consider the workload for data collection and data availability. The exact national perimeters are agreed upon with data providers (i.e., NSAs or ministries) based on the national status of HEIs. As compared with the number of tertiary education students in EUROSTAT, ETER coverage is 73% at ISCED^45^ level 5 (short diplomas), 95% at ISCED6 (bachelor) and 100% at ISCED7 (master). In terms of types of HEIs, the ETER coverage is broader than (PhD awarding) universities, to also include colleges, universities of applied sciences, and specialized institutions such as teacher training institutions, art schools, military schools, etc.

Data completeness by domain, variable and country has progressively improved over the years, and for 2020 is excellent for descriptors and geographical information (nearly 100%) and very good for students and graduates (about 95%, but some missing breakdowns); it is good for personnel data (around 80%, but lower for some breakdowns) and average for financial data (about 60%).

ETER includes several hundreds of variables and indicators, which are complemented by flags and remark fields, for a total of about 800 data fields per institution and year. Additional variables and indicators are added to the data on a rolling basis, following consultation with the ETER governance bodies – the technical support group, methodological working group and advisory board. These extensions reflect the changing needs of data users and the increasing availability of internationally comparable higher education data at the level of individual institutions.

### Variables and indicators

The ETER variables and indicators have been grouped into thirteen dimensions, as described in Table 1 below.

**Table 1:** Dimensions of ETER Data and Indicators.

Dimension	Description	Main data source
Basic Institutional Descriptors	Descriptive information on the HEI, such as legal status, foundation year, legal status, and national label, as well as external identifiers, matching HEIs in ETER with other datasets.	RISIS-OrgReg, ETER
Geographic Information	Address and geographical coordinates of the main and satellite campuses, including NUTS 2 and NUTS3 region of establishment.	RISIS-OrgReg
Expenditures	HEI expenditures (personnel expenditure, non-personnel expenditure, total current expenditure, capital expenditure), in national currency, Euro and PPP.	NSA
Revenues	Detailed breakdowns of HEI revenues (core budget, third-party funding, student fees funding, current and non-recurring revenues), in national currency, Euro and PPP.	NSA
Personnel	Academic and non-academic personnel (in both headcounts (HC) and full-time equivalents (FTE)), including breakdowns on seniority level, gender, citizenship, and fields of education (in HC only). Variables of research and teaching assistants are also included.	NSA
Education - Students	Lowest and highest degree delivered and the number of enrolled students by gender, citizenship, mobility, fields of education, age group and mode of study.	NSA
Education - Graduates	Number of graduates by gender, citizenship, mobility, fields of education, and age group.	NSA
Research	Information on research activity and the number of ISCED 8 (PhD) students and graduates by gender, citizenship, mobility, fields of education, age group and mode of study. Variables on R&D expenditures, available in national currency, Euro, and PPP.	NSA
Demographic Events	These variables identify the HEI in ETER, matching information with other datasets and information on demographic events.	RISIS-OrgReg, ETER
Erasmus Data	Incoming and outgoing Erasmus personnel and students by ISCED level. Erasmus Charter codes for the 2014–2020 and 2021–2027 periods.	Erasmus executive agency
EQAR Data	Information on whether an HEI was subject to a quality assurance process following European guidelines and reported in the DEQAR database. The number of externally quality-assured programs by ISCED level, joint programs, and cross-border programs.	DEQAR
EU-FP Project data	Participation and coordination in European Union Framework Programs by field of education along with information about the subprograms and the number of partnerships with regional or industrial partners. Information on researchers’ mobility and training cooperation supported by the Framework Programs.	EUPRO
Indicators	Indicators to characterize HEIs across dimensions such as gender balance, mobility, internationalization, and research orientation. Indicators are computed from (combinations of) data in ETER. Examples include the share of female students, the share of foreign personnel, and the share of graduates in science and technology fields.	ETER

For an up-to-date and full description of individual variable definitions, types and data sources, indicators definitions, general rules and methods of computation, as well as detailed information on flags, special codes and remarks, the reader should refer to the latest version of the ETER Handbook^43^.

### Flags, special codes and notes

Special codes conforming with guidelines for the UOE data collection are applied throughout the dataset, as—in general—no blank cells are allowed in the data collection except for the ‘Notes’ fields. The most important codes are ‘m’ (data missing) and ‘a’ (variable is not applicable for the unit of data collection).

In addition to special codes, flags are used to identify deviant cases (in terms of format accuracy, consistency, completeness, and comparability). They are accompanied by an explanation in the ‘Notes’ field providing an explanation of why the value is deviant. Examples of flags are ‘b’ for break in series due to a change in the data collection procedures, ‘de’ for a break due to a demographic event, ‘d’ to inform the user that the definition adopted in the data collection procedures departs from the ETER Handbook^43^.

## Technical Validation

**Fig. 2:**
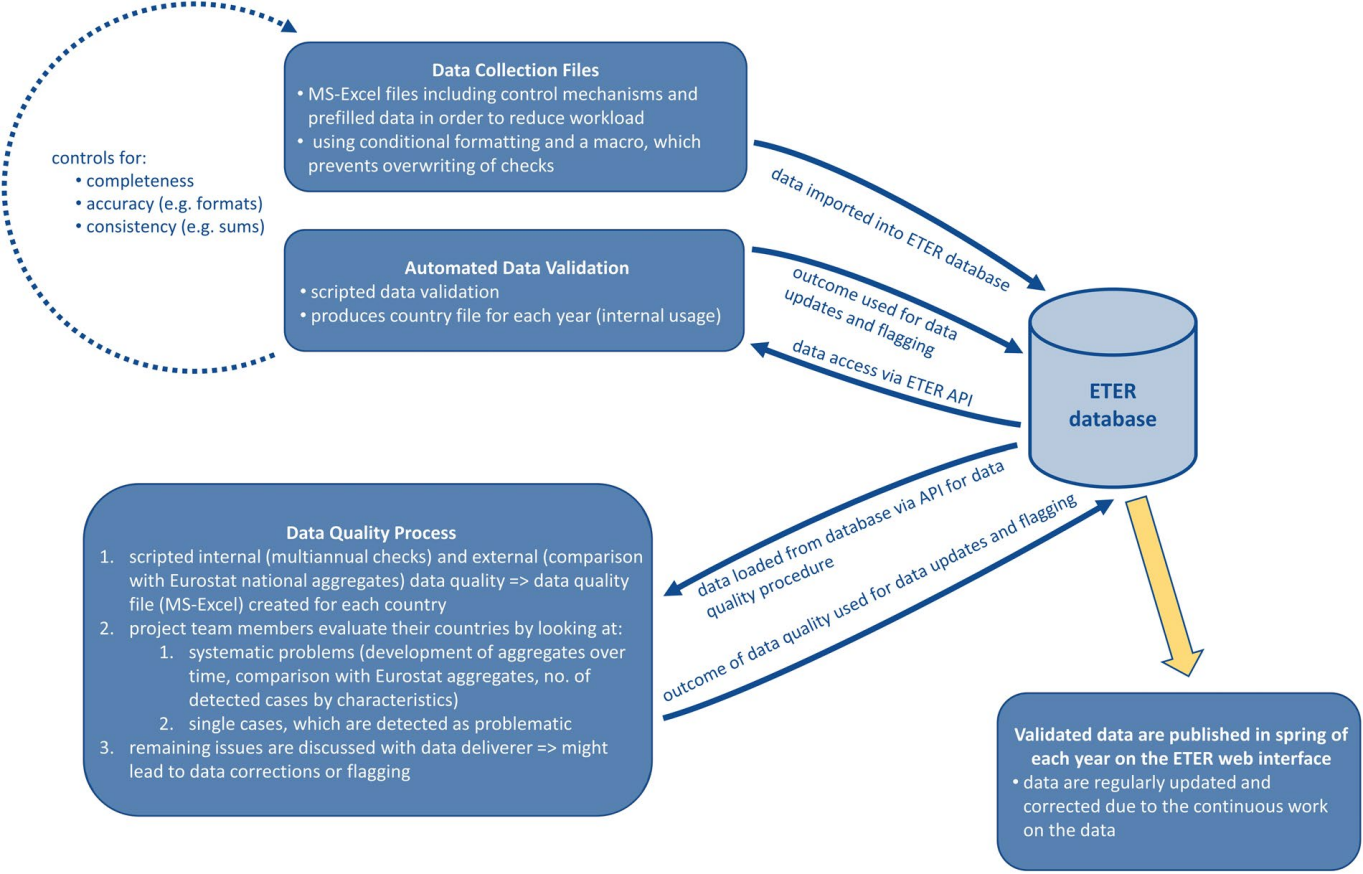
Overview of the ETER data validation and data quality process.

In order to detect possible inconsistencies among the variables delivered annually, the ETER project team developed an automated multi-level data validation and quality process (see also Fig. 2). It consists of (1) control mechanisms and prefilled data in the data collection files, (2) an automated data validation process after receiving data collection files from the data deliverer, and (3) an internal and external data quality process after the main data collection.

### Control mechanisms in the data collection files

Manual checking of data in ETER has become unfeasible due to the extensive amount of data collected. The issues were manifold and included incomplete data, accuracy problems (e.g., wrong formats), or totals not matching the sums of breakdowns. Many of those cases could be spotted automatically by simple dependency rules between variables.

To address these issues, ETER has implemented control mechanisms into the data collection files utilizing conditional formatting. Whenever a value is out of the expected range of possible values, the data deliverer is alarmed by a coloured cell. Colors show data deliverer missing values (yellow), accuracy problems (orange) or issues with sums not matching totals of breakdowns (red). Their purpose is to make data deliverers aware of unusual values and prompt them to either correct or flag them.

The control mechanisms implemented in the data collection files allowed us to significantly reduce the number of inconsistencies in the data.

### Data validation

As a second layer, data are uploaded into the database, and a script, using an API endpoint, runs on the data and produces a report for each country for internal usage. The types of checks are the same as in the data collection file (completeness, accuracy, and consistency), but the scripted data validation is more comprehensive.

The generated report per country and year shows irregularities in the data. It is used internally for examining detected cases, with each detected case is then either corrected or flagged. The data validation process is valuable for the project since it guarantees a clean dataset for ETER users.

### Data quality process

The ETER data quality process is a semi-automated process consisting of scripted internal and external quality checks and interaction of the ETER project team with the data deliverer.

*External data quality* includes a comparison of ETER and Eurostat data. Although ETER data are based on the individual level and aggregation to country totals is not recommended (because of differences in the perimeter), the project team compares aggregated ETER data to Eurostat national data. Two aspects can signify issues in the data: (1) coverage, which should not differ too much, and (2) the development of aggregated ETER data and Eurostat totals, which should be similar. Any large deviations in coverage or the development of data are examined by the project team and, if needed, reported back to NSAs to investigate the reasons.

*Multiannual checks* analyze longitudinal inconsistencies for a core set of variables, such as the total number of students and the total revenues, which are expected to evolve smoothly. The approach includes two types of control mechanisms: (1) check of discontinuity, which captures multiannual volatility by identifying large variations in the values of variables, and (2) check of variance of deltas, which allows the identification of cases with moderate variations (thus not detected) but anomalous isolated jumps in the data^46^. Detected cases, especially large deviations in one year compared to all other years for a variable and HEI, can be an indication of methodological changes, demographic events, or mistakes. Detected cases are first examined by the project team, as some deviations might be known from previous years or due to demographic events; unexplained cases are reported back to the data deliverer, who are then expected to perform further checks on the data. By experience, some of these cases are explained by simple mistakes in the data collection, such as swapping rows, while others are due to changes in data collection procedures; the former are easily corrected, while the latter are systematically flagged in the database.

## Usage Notes

A full ETER dataset dump is available on the Zenodo repository in csv format^47^. The file includes the full dataset, a list of variables and codes and the full set of metadata. The current version on Zenodo is updated to the end of April 2023, and will be updated when major changes in the dataset occur.

In addition to the Zenodo dataset, the most up to date version of the data are available at the ETER platform at http://www.eter-project.com, which allows for various options for data search, selection and export indifferent formats.

While most data are publicly available once the national authorities have given publication permission, due to national confidentiality requirements, some ETER data are available only for research purposes on the condition that individual data points are not disclosed publicly. Access to restricted data is possible by registering on the website and accepting the non-disclosure agreement.

Metadata provide essential information on methodological issues, data sources and departures from definitions and, thus, are an important complement to the dataset. The metadata for all selected countries and years can be downloaded in a separate MS-Excel file.

A guidance on how to use the ETER interface for the microdata extraction can be downloaded from the ETER website, together with full documentation and the current version of the ETER Handbook^43^. A dedicated ‘learn’ section serves as a single-entry point to all training resources for ETER users.

Once downloaded, ETER data can be imported into most currently used software for statistical analyses, including MS Excel, SPSS, Stata, and R. A dedicated section of the ETER website provides guidance on importing the data and dealing with specific issues such as encoding non-numeric codes for missing variables.

Due to the extensive size and complexity of the dataset, which also includes scattered missing variables, with different variables missing by year and country, it is recommended to use MS Excel only for basic descriptive analyses. For more complex tasks, it is strongly advised to use statistical software such as Stata and R. The use of such software facilitates scripting tasks and enables the replication of analyses by downloading the most recent version of the dataset. For cross-sectional analyses, it might be advisable to combine different years in order to maximize availability, while interpolation techniques can be used to fill in data gaps for individual years.

An important feature of ETER is the possibility of combining it with other datasets; this is straightforward for the datasets where ETER/OrgReg identifiers have been introduced, such as the Database of External Quality Assurance Reports (DEQAR; https://www.eqar.eu/qa-results/search/by-institution/), the RISIS publication dataset maintained by the University of Leiden, and the RISIS-EUPRO dataset on participations to European Framework Programs maintained by the Austrian Institute of Technology (https://rcf.risis.io/access-request/datasets). For other datasets, name matching can be performed by combining institutional names (in English and national language), institutional acronyms, country, cities, and websites. To this aim, it is possible to use the OpenRefine open source matching tool (https://openrefine.org/) using the reconciliation API service available on RISIS-OrgReg (https://www.risis2.eu/registers-orgreg/); this allows users to match their own list of organizations with OrgReg and to embed in their data the ETER/OrgReg identifiers.

The total number of data points in the current version of the dataset is about 3,500 HEIs × 300 variables × 10 years, i.e., about 10.5 m data points; this number doubles by including flags and notes fields. The total file size in CSV format is over 80 MB.

## Data Availability

The European Commission is the owner of the ETER source code under contract no. EAC 2021-0170. While the ETER infrastructure is open for public usage, the source code itself is not public.

## References

[1] Godin B (2002). The Number Makers: A Short History of International Science and Technology Statistics. Minerva.

[2] UOE. in *UOE data collection on education systems. Volume 1. Manual. Concepts, definitions, classifications* (UNESCO, OECD, Eurostat., Montreal, Paris, Luxembourg, 2013).

[3] OECD. in *Frascati Manual 2015. Guidelines for Collecting and Reporting Data on Research and Experimental Development* (OECD, Paris, 2015).

[4] OECD. in *OECD Science, Technology and Industry Scoreboard 2009* (OECD, Paris, 2009).

[5] OECD. in *Education at a Glance* (OECD, Paris, 2016).

[6] Huisman J (2000). Higher education institutions: as different as chalk and cheese. Higher Education Policy.

[7] Birnbaum, R. in *Maintaining diversity in higher education* (Jossey-Bass, San Francisco, 1983).

[8] Braun, D. & Merrien, F. X. in *Towards a New Model of Governance for Universities? A Comparative View*. (Jessica Kingsley, London, 1999).

[9] Musselin, C. in *Towards a Multiversity? Universities between Global Trends and National Traditions* (eds Krücken, G., Kosmützky, A. & Torka, M.) 63–84 (transcript, Bielefeld, 2007).

[10] Kyvik S (2004). Structural changes in higher education systems in Western Europe. Higher education in Europe.

[11] Ferlie E, Musselin C, Andresani G (2008). The steering of higher education systems: A public management perspective. Higher education.

[12] Brunsson N, Sahlin-Andersson K (2000). Constructing organizations: The example of the Public Sector Reform. Organization Studies.

[13] Bonaccorsi, A. in *Knowledge, Diversity and Performance in European Higher Education. A Changing Landscape* (Edward Elgar, Cheltenam, 2014).

[14] Lepori B (2022). The heterogeneity of European Higher Education Institutions: a configurational approach. Studies in Higher Education.

[15] Lepori B, Borden VM, Coates H (2022). Opportunities and challenges for international institutional data comparisons. European Journal of Higher Education.

[16] Bonaccorsi A, Daraio C, Lepori B, Slipersæter S (2007). Indicators on individual higher education institutions: addressing data problems and comparability issues. res eval.

[17] Daraio C (2011). The European university landscape. Research policy.

[18] Lepori B (2020). A register of public-sector research organizations as a tool for research policy studies and evaluation. Research Evaluation.

[19] Heller-Schuh B, Lepori B, Neuländtner M (2020). Mergers and acquisitions in the public research sector. Toward a comprehensive typology. Research Evaluation.

[20] Lepori, B., Bornmann, L. & de Moya Anegón, F. Measuring university size. A comparison of Academic personnel versus Scientific Talent Pool data. *Quantitative Science Studies* (2023).

[21] Lepori, B., Seeber, M. & Bonaccorsi, A. Competition for talent. Country and organizational-level effects in the internationalization of European higher education institutions. *Research Policy*. 10.1016/j.respol.2014.11.004 (2014).

[22] Seeber M, Meoli M, Cattaneo M (2020). How do European higher education institutions internationalize. Studies in Higher Education.

[23] Afonso A (2016). Varieties of academic labor markets in Europe. PS: Political Science & Politics.

[24] Baltaru R, Soysal YN (2018). Administrators in higher education: organizational expansion in a transforming institution. Higher Education.

[25] Avenali A, Daraio C, Wolszczak-Derlacz J (2023). Determinants of the incidence of non-academic staff in European and US HEIs. Higher Education.

[26] Gadar L, Kosztyan ZT, Telcs A, Abonyi J (2020). A multilayer and spatial description of the Erasmus mobility network. Scientific Data.

[27] Bruni R, Catalano G, Daraio C, Gregori M, Moed HF (2020). Studying the heterogeneity of European higher education institutions. Scientometrics.

[28] Huisman J, Lepori B, Seeber M, Frølich N, Scordato L (2015). Measuring institutional diversity across higher education systems. Research Evaluation.

[29] Bonaccorsi A, Cicero T, Haddawy P, Hassan S (2017). Explaining the transatlantic gap in research excellence. Scientometrics.

[30] Lepori, B., Geuna, A. & Mira, A. Scientific output scales with resources. A comparison of US and European universities. *PloS one* **14**(10) 10.1371/journal.pone.0223415 (2019). 10.1371/journal.pone.0223415 PMC6793846 31613903

[31] Agasisti, T. in *Handbook on Public Sector Efficiency* (eds Afonso, A., Tovar Jalles, J. & Venâncio, A.) 274-290 (Edward Elgar Publishing, 2023).

[32] Bonaccorsi, A., Barin, L., Bellingheri, P., Biagi, F. & Barrioluengo, M. S. Is higher education more important for firms than research? Disentangling university spillovers. *Journal of Technology Transfer* (2023).

[33] Hunady J, Orviska M, Pisar P (2019). What matters: the formation of university spin-offs in Europe. Business Systems Research: International journal of the Society for Advancing Innovation and Research in Economy.

[34] Callado-Muñoz FJ, Utrero-González N (2019). Integration in the European higher education area: the case of military education. Defence Studies.

[35] de la Torre, E. M. & Perez-Esparrells, C. in *Collaboration, Communities and Competition* (ed Dent, S., Lane, L., Strike, T.) 51–71 (Brill, 2017).

[36] Lambrechts, A. A., Cavallaro, M. & Lepori, B. The European Universities initiative: between status hierarchies and diversity. (2023). 10.35542/osf.io/qp9rm 10.1007/s10734-023-01167-w PMC11461708 39391862

[37] Català-Oltra L, Martínez-Gras R, Penalva-Verdú C (2023). The Use of Languages in Digital Communication at European Universities in Multilingual Settings. *International Journal of Society*. Culture & Language.

[38] Artero López JM, Borra Marcos C, Gómez-Álvarez Díaz R (2020). Education, inequality and use of digital collaborative platforms: The European case. The Economic and Labour Relations Review.

[39] Crosier, D., Birch, P., Davydovskaia, O., Kocanova, D. & Parveva, T. Modernisation of Higher Education in Europe: Academic Staff–2017. Eurydice Report. *Education, Audiovisual and Culture Executive Agency, European Commission* (2017).

[40] Poelman, H. & Dijkstra, L. Access to universities in the EU: A regional and territorial analysis. https://ec.europa.eu/regional_policy/en/information/publications/regional-focus/2018/access-to-universities-in-the-eu-a-regional-and-territorial-analysis (2018).

[41] Jongbloed, B., McGrath, C., de Boer, H. F. & de Gayardon, A. Final report of the study on the state and effectiveness of national funding systems of higher education to support the European universities initiative. Volume I. *Directorate-General for Education, Youth, Sport and Culture Youth, Education and Erasmus+*, (2023).

[42] Barrioluengo, M. S. & Flisi, S. Student mobility in tertiary education: institutional factors and regional attractiveness. *Publications Office of the European Union, Luxembourg. EUR* **28867** (2017).

[43] ETER project (2023). Zenodo.

[44] Lepori, B. *et al*. in *Implementation and further development of the European Tertiary Education Register* (Publications Office of the European Union, Brussels, 2020).

[45] UNESCO. International Standard Classification of Educational Degrees (ISCED) (2023).

[46] Daraio C (2020). A tailor-made data quality approach for higher educational data. Journal of Data and Information Science.

[47] ETER (2023). Zenodo.

